# Carotenoids are used as regulators for membrane fluidity by *Staphylococcus xylosus*

**DOI:** 10.1038/s41598-019-57006-5

**Published:** 2020-01-15

**Authors:** Waldemar Seel, Denise Baust, Dominik Sons, Maren Albers, Lara Etzbach, Janina Fuss, André Lipski

**Affiliations:** 1 Rheinische Friedrich-Wilhelms-Universität Bonn, Institute of Nutritional and Food Science, Food Microbiology and Hygiene, 53115 Bonn, Germany; 2 Rheinische Friedrich-Wilhelms-Universität Bonn, Institute of Nutritional and Food Science, Molecular Food Technology, 53115 Bonn, Germany; 3 Max Planck-Genome-Centre Cologne, 50829 Cologne, Germany; 4 Present Address: Institute of Clinical Molecular Biology, Kiel University (CAU)/University Hospital Schleswig Holstein, 24105 Kiel, Germany

**Keywords:** Cellular microbiology, Bacteriology

## Abstract

Carotenoids are associated with several important biological functions as antenna pigments in photosynthesis or protectives against oxidative stress. Occasionally they were also discussed as part of the cold adaptation mechanism of bacteria. For two *Staphylococcus xylosus* strains we demonstrated an increased content of staphyloxanthin and other carotenoids after growth at 10 °C but no detectable carotenoids after grow at 30 °C. By *in vivo* measurements of generalized polarization and anisotropy with two different probes Laurdan and TMA-DPH we detected a strong increase in membrane order with a simultaneous increase in membrane fluidity at low temperatures accompanied by a broadening of the phase transition. Increased carotenoid concentration was also correlated with an increased resistance of the cells against freeze-thaw stress. In addition, the fatty acid profile showed a moderate adaptation to low temperature by increasing the portion of anteiso-branched fatty acids. The suppression of carotenoid synthesis abolished the effects observed and thus confirmed the causative function of the carotenoids in the modulation of membrane parameters. A differential transcriptome analysis demonstrated the upregulation of genes involved in carotenoid syntheses under low temperature growth conditions. The presented data suggests that upregulated synthesis of carotenoids is a constitutive component in the cold adaptation strategy of *Staphylococcus xylosus* and combined with modifications of the fatty acid profile constitute the adaptation to grow under low temperature conditions.

## Introduction

Carotenoids represent a large group and comprise at least 800 described compounds^1,2^ which are synthesized from isoprene units by phototrophic and chemoorganotrophic prokaryotes, plants, fungi, and algae^3^. Most carotenoids consist of eight isoprene units resulting in a C40 backbone and usually display β-cyclisation^1,4^. Only a few chemoorganotrophic bacteria are capable of producing C30, C45 or C50 carotenoids^4,5^. Such a rare C30 carotenoid is produced by staphylococci^6,7^. As lipophilic compounds, carotenoids are located in the cell membrane, but their orientation inside the membrane may vary depending on their chemical structure and on the thickness of the membrane^8,9^. They perform important biological functions such as light harvesting as antenna pigments^10,11^, provide protection against oxidative stress^5,12^, provide protection from ultraviolet radiation^13,14^, and stabilization of pigment proteins^15^. In addition to the functions mentioned above, the involvement of carotenoids in cold adaptation was suspected^16–18^. We assumed that carotenoids might have a similar function in regulating membrane fluidity as sterols such as cholesterol or ergosterol in eukaryotic cells. They increase membrane order with concurrent maintenance of lateral lipid motility, which results in a liquid-ordered membrane state^19^. Similar mechanisms were previously described for the bacterial sterol-like hopanoids^20,21^ and isoprenoid quinones^22^. *In vitro* studies with artificial membrane vesicles already showed regulatory effects on membrane fluidity by carotenoids^23,24^. The species *Staphylococcus xylosus* is well known for temperature dependent pigmentation, a characteristic which is already mentioned in the species description^25^. In line with this description we found strong pigmentation at a growth temperature of 10 °C but no pigmentation at 30 °C for strain *S. xylosus* J70, isolated from a food sample. Therefore, we tested the hypothesis that carotenoids could act as a cholesterol-like analogue improving membrane properties under low temperature growth conditions in *S. xylosus* as a model organism. As a reference, type strain DSM 20266 ^T^ was also analysed. The methods applied in this work, allowed us to detect changes in membrane order and lipid mobility *in vivo* by measuring general polarization and anisotropy of whole cells, respectively.

## Results

### Fatty acid profiles and isoprenoid quinone content

Fatty acid profiles were established for both *S. xylosus* strains at 30 °C and 10 °C growth temperature at the late exponential phase. The strains showed an iso/anteiso fatty acid profile and the adaptive response to low temperature is mainly based on increase of the portion of anteiso fatty acids (Table 1). The ΔWAMT-values were calculated to derive the extent of fatty acid-dependent temperature adaptation^22^. Both isolates showed a similar alteration in fatty acid composition resulting in a ΔWAMT of about 6 °C. The analyses of NaCl-supplemented cultures of strain J70 revealed only small changes in fatty acid composition caused by salt supplementation (Table 1). This resulted in a slightly lower ΔWAMT-value of 5.2 °C.

**Table 1:** Fatty acid composition and total menaquinone content of *S. xylosus* strains related to growth temperature.

Strain	DSM 20266 ^T^		J70		J70 + 5% NaCl	
Growth temperature Fatty acid (%)	10 °C	30 °C	10 °C	30 °C	10 °C	30 °C
14:0 iso	0.6 ± 0.0	5.0 ± 0.3	0.5 ± 0.1	5.9 ± 0.2	2.0 ± 0.3	4.0 ± 0.3
14:0	n.d.	0.7 ± 0.1	n.d.	n.d.	n.d.	n.d.
15:0 iso	9.4 ± 0.1	18.2 ± 1.4	13.9 ± 0.3	19.0 ± 0.3	17.4 ± 1.6	18.7 ± 1.1
15:0 anteiso	71.3 ± 1.2	56.2 ± 1.7	68.0 ± 2.4	53.3 ± 0.7	70.5 ± 2.8	58.1 ± 4.2
16:0 iso	1.1 ± 0.1	4.4 ± 0.3	2.4 ± 0.1	5.5 ± 0.3	2.6 ± 0.5	3.8 ± 0.2
16:0	n.d.	1.9 ± 0.2	n.d.	1.8 ± 0.0	n.d.	1.7 ± 0.2
17:0 iso	1.5 ± 0.1	3.6 ± 0.1	2.6 ± 0.1	4.3 ± 0.1	1.9 ± 0.5	4.0 ± 0.7
17:0 anteiso	12.4 ± 0.2	3.8 ± 0.6	10.1 ± 0.4	3.6 ± 0.0	4.5 ± 0.6	3.9 ± 0.5
18:0	1.2 ± 0.9	4.2 ± 2.2	2.6 ± 0.2	3.7 ± 0.1	0.7 ± 0.1	4.1 ± 1.4
20:0	2.5 ± 0.3	1.4 ± 0.2	n.d.	3.8 ± 1.0	n.d.	1.7 ± 0.4
WAMT (°C)	31.2	37.4	32.3	38.8	31.9	37.1
ΔWAMT (°C)	6.2		6.5		5.2	
Menaquinone concentration (nmol/g _cell wet weight_)	217 ± 10	250 ± 5	254 ± 14	296 ± 8	n.a.	n.a.

Temperature adaptations are presented as weighted average melting temperature (WAMT) of the profiles (n.d.: not detected; n.a.: not analysed). Numbers following ± sign are standard deviations.

In both *S. xylosus* strains, three different menaquinones were detected. Major menaquinone was MK-7 with a share of about 90%. Two minor menaquinones were MK-6 and MK-8 in approximately equal proportions (Table 1). The low temperature cultivation resulted in a reduction in menaquinone content of about 15–20% in both strains.

### Carotenoid analysis

Colonies of both *S. xylosus* strains showed no visible pigmentation at 30 °C growth temperature. This was confirmed by chromatography analysis, which showed a carotenoid concentration below the detection limit of 0.2 µg/g_cell dry weight_ (Table 2). Low-temperature incubation resulted in a strong orange pigmentation of cell pellets and colonies of strain J70 and DSM 20266^T^. Total carotenoid content was significantly higher in the type strain with 450 µg/g_cell dry weight_ compared to the food related strain J70 with 170 µg/g_cell dry weight_. Mass spectrometry coupled HPLC detected staphyloxanthin or staphyloxanthin-like carotenoids as major carotenoids in strain J70 (85%) and DSM 20266 ^T^ (90%). Unlike staphyloxanthin, the three staphyloxanthin-like compounds were characterized by a fatty acid different from 15:0 anteiso. In strain J70, the staphyloxanthin-like compounds share accounted for 30% and was clearly lower than in strain DSM 20226 ^T^ with about 60% (Table 2). The precursor all-*trans*-4,4′-diaponeurosporeate also represented a minor carotenoid with a proportion of 15% in strain J70 and 7% in strain DSM 20266^T^. *Cis*-isomers of some compounds (Table 2, values are in brackets) were also detected with a share of 0.5–3%.

**Table 2:** Carotenoid composition in percent of *S. xylosus* strains J70 and DSM 20266^T^ related to growth temperature.

Strain		DSM 20266 ^T^		J70	
Carotenoid (%)	t_R_ (min) UV	10 °C	30 °C	10 °C	30 °C
all-*trans*-4,4′-diaponeurosporeate	3.199	6.7	n.d.	15.0	n.d.
staphyloxanthin-like	4.003	2.5	n.d.	4.0	n.d.
all-*trans*-staphyloxanthin	4.691 (4.318)	32.5 (0.4)	n.d.	50.5 (0.9)	n.d.
staphyloxanthin-like	5.638 (5.165)	21.8 (1.8)	n.d.	13.2 (1.7)	n.d.
staphyloxanthin-like	6.800 (6.227)	23.7 (2.8)	n.d.	11.3 (1.4)	n.d.
staphyloxanthin-like	7.991 (7.475)	6.7 (1.2)	n.d.	2.0 (0.0)	n.d.
Total carotenoid concentration (µg/g_cell dry weight_)		451.4 ± 20.0	n.d.	169.0 ± 11.1	n.d.

Carotenoid data obtained by HPLC-DAD-APCI-MS^*n*^. Corresponding *cis*-Peaks shown in brackets. Total carotenoid concentration displayed in (µg/g_cell dry weight_) (n.d.: not detected). Numbers following ± sign are standard deviations, t_R_ = retention time.

### Transcriptome profiling of differentially expressed genes at low growth temperatures

The genome sequence of *S. xylosus* J70 has a total size of 2,820,948 bp and a GC content of 32.7% (GenBank WGS Accession number SSMH00000000). The SPAdes assembler predicted 2660 genes. The raw data of mRNA sequencing has been submitted to the NCBI Sequence Read Archive (SRA) with the accession number PRJNA532330.

The *in situ* response of *S. xylosus* J70 revealed an extensive change in gene expression at cold growth temperature. This resulted in 514 up-regulated genes, 502 down-regulated genes and 1568 genes with no temperature-dependent effect on expression level. We could demonstrate that the cold-induced pigmentation of strain J70 is accompanied by the up-regulation of carotenoid biosynthesis associated operon *crtOPQMN*. On average, we observed a 4-fold change in the individual genes involved in carotenoid synthesis (Table 3). As a reference data set for up-regulated genes under low temperature conditions, we used the genes *cudBACT* responsible for synthesis of the cryoprotectant betaine and the cold shock protein gene of the *cspA*-family, which showed 2-fold to 22-fold upregulation upon low temperature incubation. Genes associated with menaquinone synthesis (*menA-F*) did not show significant change, on average. A set of six heat shock protein (Hsp) coding genes showed a clearly reduced expression at low temperatures. Genes *groEL* and *groES* showed a remarkable 5-fold decrease in reads, considering their function ensures correct protein folding. As reference genes with no expected change of expression level we listed three housekeeping genes^26^ which showed no temperature-dependent change of the expression level.

**Table 3:** Transcriptional upregulation for genes of *S. xylosus* J70 grown at 10 °C compared to growth at 30 °C.

Gene ID	Gene name	Description	Fold change
**Carotenoid synthesis**			
1195	*crtN*	Dehydrosqualene desaturase	4.4
1196	*crtM*	Dehydrosqualene synthase	4.1
1197	*crtQ*	4,4-diaponeurosporenoate glycosyltransferase	4.1
1198	*crtP*	Diapolycopene oxygenase	3.9
1199	*crtO*	Glycosyl-4,4-diaponeurosporenoate acyltransferase	4.4
**Betaine synthesis (Cryo protectant)**			
1019	*cudB*	Oxygen-dependent choline dehydrogenase	7.6
1020	*cudA*	Glycine betaine aldehyde dehydrogenase	7.0
1021	*cudC*	Glycine betaine synthesis regulator	21.6
1022	*cudT*	Choline transporter	4.0
**Cold shock protein**			
1434	*cspA*-family	Cold shock protein	2.1
**Menaquinone synthesis**			
48	*menE*	2-succinylbenzoate–CoA ligase	1.0
49	*menC*	O-succinylbenzoic acid synthetase	0.9
838	*menB*	1,4-dihydroxy-2-naphthoyl-CoA synthase	0.9
839	*menH*	2-succinyl-6-hydroxy-2,4-cyclohexadiene-1-carboxylate synthase	1.2
840	*menD*	2-succinyl-5-enolpyruvyl-6-hydroxy-3-cyclohexene-1-carboxylic-acid synthase	1.2
841	*menF*	Isochorismate synthase	1.7
842	*menA*	1,4-dihydroxy-2-naphthoate octaprenyltransferase	0.6
**Housekeeping-genes (control)**			
1262	*gyrB*	DNA gyrase subunit B	1.1
1261	*gyrA*	DNA gyrase subunit A	1.2
2221	*secA*	Preprotein translocase subunit	1.1
**Heat shock proteins (Hsps)**			
2397	*groES*	Co-chaperone GroES (Hsp10)	0.2
2398	*groEL*	Chaperonin GroEL (Hsp60)	0.2
245	*hrcA*	heat-inducible transcription repressor HrcA	0.4
246	*grpE*	Co-chaperone for DnaK	0.5
247	*dnaK*	Molecular chaperone DnaK (Hsp70)	0.4
248	*dnaJ*	Molecular chaperone DnaJ (Hsp40)	0.6

### Effects of carotenoids on membrane order and fluidity

**Figure 1:**
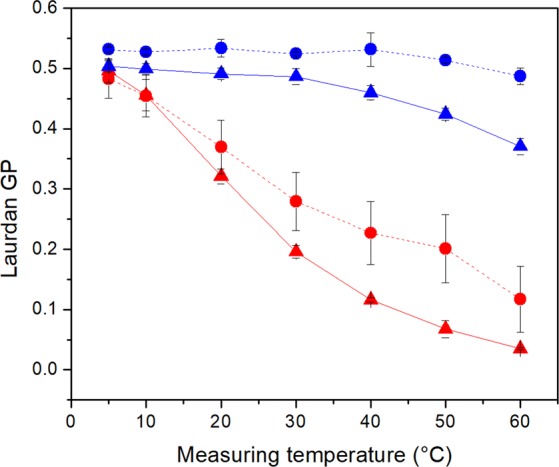
Temperature-dependent membrane order analysed by Laurdan GP-values of *S. xylosus* strains DSM 20266 ^T^ (●) and J70 (▲) at 30 °C (red) and 10 °C (blue) incubation temperature. Data represent mean values (n = 3), error bars represent standard deviations.

Laurdan GP values were measured in order to determine differences in membrane rigidity between cells grown at 30 °C und 10 °C. The data shown in Fig. 1 indicate a distinct discrepancy between cells with and without carotenoids. Both strains, grown at 30 °C, showed a steady decrease of their GP value as the measuring temperature increased. In contrast, we were able to obtain a consistently high Laurdan GP value over a larger temperature range in cold incubated cells. In strain J70, a GP value of 0.5 from 5 °C to 30 °C indicated a constant high cell membrane order within this temperature range. A further increase in measurement temperature resulted in a steady GP value reduction from 0.5 to 0.37 indicating a decrease in membrane order above 30 °C. In strain DSM 20266 ^T^ we observed a slightly higher membrane order with a GP value of 0.53 from 5 °C to 40 °C. A slight decrease in membrane order occurred at higher measuring temperatures resulting in a final GP value of 0.48. This implies a higher degree of cell membrane organization in strain DSM 20266 ^T^ compared to strain J70. This correlates also with the higher carotenoid content of strain DSM 20266 ^T^ in comparison to isolate J70 (Table 2).

**Figure 2:**
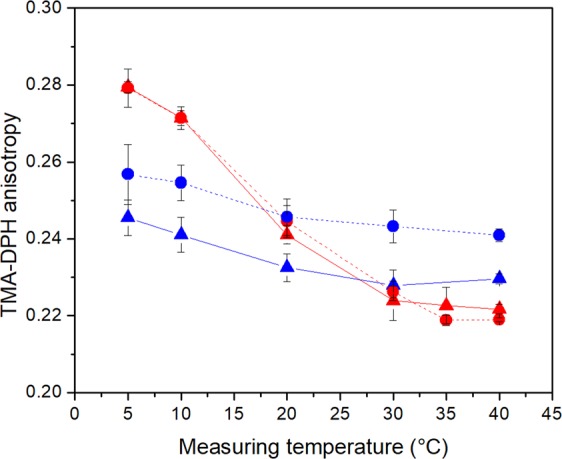
Temperature-dependent membrane fluidity analysed by TMA-DPH anisotropy values of *S. xylosus* DSM 20266 ^T^ (●) and J70 (▲) at 30 °C (red) and 10 °C (blue) incubation temperature. Data represent mean values (n = 3), error bars represent standard deviations.

We measured TMA-DPH anisotropy to determine the lateral diffusion capability of the cell membranes, which is equivalent to membrane fluidity. The results, seen in Fig. 2, revealed a similar behavior of both tested strains with a clear impact of carotenoids formed at low temperatures. *S. xylosus* cells grown at 30 °C with no carotenoid production showed high anisotropy values of about 0.28 indicating low membrane fluidity. TMA-DPH anisotropy then steadily decreased with higher measuring temperatures down to value of around 0.22. Strain J70 achieved complete phase transition to a fluid-like membrane at 30 °C, which is about 5 °C lower than the type strain DSM 20266 ^T^. *S. xylosus* grown at 10 °C, with carotenoid formation, showed a significantly smaller anisotropy change over the entire measuring range, which indicates an elevated cell membrane stability and a broad transition phase. At 5 °C, anisotropy values of 0.255 for strain DSM 20266 ^T^ and 0.245 for strain J70 were considerably lower than for the carotenoid-free samples (0.28) indicating a higher and therefore a more beneficial membrane fluidity. On the other hand, anisotropy values of the carotenoid-containing samples only decreased slightly over the measuring range with final values of 0.24 for strain DSM 20266 ^T^ and 0.23 for strain J70 resulting in less fluid membranes at higher temperatures compared to the carotenoid-free samples.

### Modification of physical membrane parameters by carotenoid suppression

**Figure 3:**
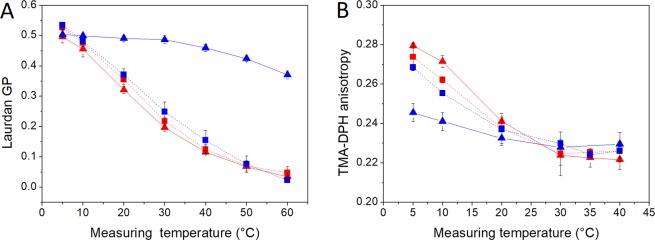
Effect of carotenoid synthesis inhibition on Laurdan-GP (**A**) and TMA-DPH (**B**) anisotropy values of *S. xylosus* J70 (▲) and J70 cultures supplemented with 5% NaCl (■) at 30 °C (red) and 10 °C (blue) incubation temperature. Data represent mean values (n = 3), error bars represent standard deviations.

To verify the effect of carotenoids on cell membrane order and fluidity we reduced their concentration at low growth temperatures by suppression of carotenoid synthesis. Synthesis was suppressed by the addition of sodium chloride according to a report of Fong *et al*.^27^. They reported the correlation between sodium chloride concentration and carotenoid synthesis for an *Arthrobacter* strain. We found a similar correlation for the *S. xylosus* strains analysed in this study. We tested both strains with additional NaCl supplementations of 2.5%, 5%, and 10%. We observed only a minor reduction in cell pigmentation at 2.5% NaCl supplementation. At 10% NaCl addition cells of both strains were no longer pigmented but the high salt stress had a negative impact on growth. Consequently, these two concentrations were not appropriate for inhibition of carotenoid synthesis. The 5% NaCl supplementation achieved an almost complete inhibition of carotenoid synthesis in strain J70 at low growth temperatures. This allowed us to verify the effect of carotenoids on biophysical cell membrane parameters under same growth temperature. At first, we analysed possible impact of the NaCl supplementation on the fatty acid proportions. We could demonstrate that NaCl supplementation had only a minor effect on the fatty acid composition (Table 1). The calculated WAMT values indicated a similar degree of fatty acid-depended cold adaptation, regardless of the salt concentrations. Furthermore, we did not observe any impairment of growth rates for growth with 5% NaCl. The decreased carotenoid content showed a clear impact on membrane order and fluidity (Fig. 3). The strong induction of membrane order, caused by high carotenoid concentrations, was completely lost, as shown by the similar decrease in Laurdan GP values of the carotenoid-free samples at both growth temperatures. TMA-DPH anisotropy values showed conformity of the phase transition pattern between carotenoid-free samples grown at 30 °C and 10 °C cultures supplemented with NaCl. This resulted in a loss of membrane fluidity at low temperatures. NaCl supplementation of cultures grown at 30 °C confirmed that the NaCl addition itself had no effect on membrane order and fluidity.

### Resistance against temperature stress

**Figure 4:**
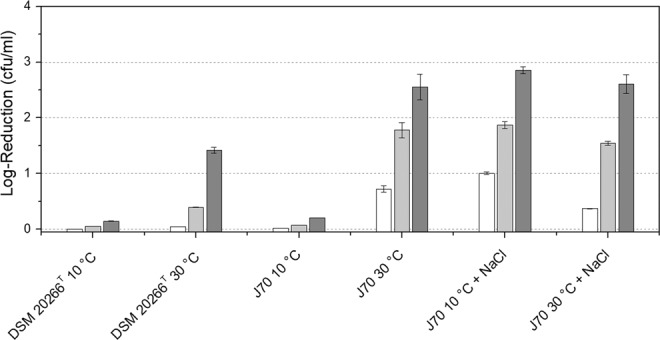
Logarithmic reduction of viable cell count of both *S. xylosus* strains after one (white), two (light grey), and three (grey) freeze-thaw cycles (each 24 h). Data represent mean values (n = 3), error bars represent standard deviations.

Temperature stress test demonstrated the beneficial impact of carotenoids on cell resistance to temperature stress. Each *S. xylosus* strain showed a clear log reduction in viable cell count after growth at 30 °C in comparison to the cultures grown at 10 °C (Fig. 4). For cells grown at 30 °C, viable cell count was reduced gradually after every freeze-thaw cycle up to a maximum of 1.5 log units in strain DSM 20266 ^T^ and 2.5 log units in strain J70 after the final freeze-thaw step. Almost no reduction (<log 0.2) in viable cell count was detected for cultures grown at 10 °C. In contrast, cultures grown at 10 °C but with carotenoid synthesis suppressed by NaCl supplementation, high log reduction rates of 2.6–2.9 log units were demonstrated. These reduction rates were equal to those cultures grown at 30 °C without carotenoid formation (Fig. 4). NaCl supplementation itself had no clear impact on log reduction rates. *S. xylosus* J70 grown at 30 °C showed similar log reduction rates independent of the presence of NaCl.

## Discussion

The increased production of carotenoids under low temperature growth conditions was observed for several bacterial species including *Staphylococcus xylosus* frequently in the past^25,27,28^. Some authors associated this phenomenon with adaptation mechanisms to low growth temperature but until now, there was no experimental proof for the involvement of carotenoids in the modification of membrane fluidity at low temperature conditions and thus in cold adaptation.

In this work, we analysed two *S. xylosus* strains with an apparent cold induced carotenoid synthesis. Our central focus was to examine the hypothesis whether carotenoids could represent a fatty acid-independent cold adaptation of the cell membrane, as previously described for isoprenoid quinones in *Listeria monocytogenes* by Seel *et al*.^22^. We detected significant changes in the fatty acid profiles and carotenoid content between 30 °C and 10 °C growth temperature in both strains. Membrane fluidity as well as membrane order were strongly modified by high carotenoid concentrations (Figs. 1, 2) and showed a positive effect on cell survivability after freeze-thaw stress (Fig. 4).

Both tested *S. xylosus* strains showed a temperature dependent response of their fatty acid profiles (Table 1) which is in accord with known adaptation mechanisms^29^. Adaptation mechanism was mainly based on a shift from iso-branched fatty acids to anteiso-branched fatty acids, which have considerably lower melting temperatures. Fatty acid-dependent cold adaptation was similar in both tested strains. The extent of fatty acid modification, calculated with the help of ΔWAMT-values, were almost identical with a ΔWAMT-value of 6.2 °C for strain DSM 20266 ^T^ and 6.5 °C for strain J70. We ran a HPLC-assisted quinone analysis, in order to exclude the influence of isoprenoid quinones on the fatty acid-independent cold adaptation, as previously shown for some *L. monocytogenes* strains^22^. Menaquinone concentration decreased by approximately 15% in both strains after temperature reduction without changes of the menaquinone profile (Table 1). We deduced therefrom that isoprenoid quinones were not mainly involved in fatty acid-independent cold adaptation.

Carotenoids form a very large and wide-ranging group of compounds that has already been well studied. Their localization and orientation within the cell membrane is well known^8,9^. Several functions were attributed to carotenoids, including light harvesting as antenna pigments in photosynthesis systems^10,11^, the reduction of oxidative stress^12,13^ or as a virulence factor in *Staphylococcus aureus*^30^. In this study, we were now able to add another functionality to carotenoids. The data presented in this study points to a function as a further fatty acid-independent cold adaptation mechanism of cell membranes similar to sterols in eukaryotes. Food-related strain J70 as well as type strain DSM 20266 ^T^ showed pigmentation only at 10 °C growth temperature. Carotenoid quantification confirmed that carotenoids were only formed at low temperatures. At 30 °C growth temperature at the exponential phase for both *S. xylosus* strains carotenoid concentration was below the detection limit of 0.2 µg/g_cell dry weight_. Cold incubation conditions led to a strong induction of carotenoid synthesis resulting in 169 µg/g_cell dry weight_ total carotenoid content in strain J70 and a two and half times higher amount of 451 µg/g_cell dry weight_ in strain DSM 20266 ^T^. At least 85% of the carotenoids detected were staphyloxanthin or staphyloxanthin-like derivatives. MS data indicated that the staphyloxanthin-like compounds differ only in the esterified fatty acid at the carbohydrate residue. Staphyloxanthin was described as a 15:0 anteiso fatty acid in *S. aureus*^31^. Higher yields of carotenoids at low temperatures were also reported for *Micrococcus roseus* before^28^.

We were able to confirm cold induction of carotenoid synthesis by differential transcriptome analysis, which revealed an upregulation of genes involved in carotenoid synthesis. This demonstrated that increase of carotenoid concentration is a part of the controlled response to low growth temperatures (Table 3). On average, we observed a 4-fold increase of the *crtOPQMN* genes involved in carotenoid synthesis. The specific reaction steps for the respective enzymes are described in detail for *S. aureus*. First, CrtM and CrtN enzymes catalyze the formation of the first yellow-colored pigment 4,4′-diaponeurospoerene through condensation of two farnesyl diphosphate molecules and successive dehydrogenation steps^32^. Enzymes CrtP, CrtQ and CrtO are involved in the subsequent oxidation, glycosylation and esterification steps to form the final pigment staphyloxanthin^31^. The postulated sixth gene *aldH* involved in carotenoid synthesis could not be clearly identified in the genome sequence obtained in our study^33^. The cold stress of the cells was further illustrated by increased expressions of the betaine synthesis genes and cold shock protein A (CspA, Table 3) as well as the reduced expression of heat shock proteins. The relatively mild increase in reads of *cspA* could be explained by possible increase of protein stabilization at low temperature and therefore induction based on increased mRNA half-life^34,35^. The individual genes of betaine synthesis were induced 4–21 times stronger at low temperatures. Various publications confirm an important role of compatible solutes as part of the cold adaptation in mesophilic bacteria^36,37^. Heat shock proteins are often chaperones that facilitate correct folding of proteins damaged by high temperatures. Two of this major protein folding mechanisms (GroEL/ES and DnaK/J) showed medium to strong reduction of the corresponding genes (Table 3 ^38^). This also demonstrates a possible energy saving potential in cell maintenance through more efficient biosynthesis, which might also lead to better cell yields at low temperature^39^. Overall we could not observe significant changes in expression of genes involved in menaquinone synthesis^40^. This was in accord with the minor changes in menaquinone content at different growth temperatures (Table 1).

As early as 1974, Nes^16^ suspected that carotenoids were involved in cold adaptation. As a result, carotenoid-producing Antarctic bacteria in particular came into focus^18,41,42^. Later, this assumption was specified and it was assumed that carotenoids, similar to eukaryotic sterols, are located in the cell membrane and influence membrane properties^8,17,23,43^. Further evidences for the involvement of carotenoids in cold adaptation was also provided for the cyanobacterium *Synechocystis*^44^. The next step was to extract carotenoids and conduct *in vitro* studies with artificial lipid vesicles. Subczynski *et al*.^23^ showed that polar carotenoids could reduce membrane fluidity above the transition temperature and had a fluidizing effect below it, a mechanism also known from cholesterol. The effect on the membrane fluidity was mainly impacted by polar carotenoids^24^. By TMA-DPH dependent anisotropy measurements we were able to confirm this impact of polar carotenoids on the membrane fluidity of whole living cells with a more complex lipid composition (Fig. 2). The incubation at 30 °C restrained the carotenoid synthesis of both strains exhibiting an almost complete phase transition, recognizable by the sigmoidal curve of the measured anisotropy in a temperature range from 5–40 °C. Characteristically, constant anisotropy values at the limits of the measuring range indicate the complete phase transition of bio-membranes from the gel-like solid state (high anisotropy) to the liquid-crystalline fluid state (low anisotropy). The cultures grown at 10 °C with high carotenoid content showed highly broadened phase transitions in both strains, recognizable by the almost linear relationship of TMA-DPH anisotropy and measured temperature. This indicated significantly more fluid membranes below 20 °C and lower fluidity above 30 °C. The change in anisotropy with a value of 0.02 was approximately the same for both strains. However, strain DSM 20266 ^T^, with the significantly higher carotenoid content, had on average higher anisotropy values than strain J70. Nevertheless, membrane fluidity was almost constant over the entire temperature range.

Membrane order as a second membrane characteristic was measured by Laurdan-GP and should provide a further insight on the membrane-carotenoid-interaction in whole cells^45^. Cultures without detectable carotenoid yields showed a steadily decrease in GP-value and therefor a constant decrease in membrane order with increasing temperature (Fig. 1, cultures grown at 30 °C). Carotenoids had a rigidifying effect on the cell membrane (cultures grown at 10 °C). Low temperatures led to constantly high GP-values up to 30 °C (strain J70) and 40 °C (strain DSM 20266^T^), respectively. The higher carotenoid content of the type strain correlated with a stronger ordering effect. Higher measuring temperatures led to slow decrease in GP-values and thus to a reduction in membrane order. The rigidifying effect of carotenoids was also previously described for zeaxanthin in artificial lipid vesicles^24^. It is also in accord with earlier reports of Harris *et al*.^45^, Sáenz *et al*.^20,21^ and Seel *et al*.^22^, who showed that membrane is strongly ordered and condensed by storage of other membrane associated lipophilic substances such as cholesterol, hopanoids, and isoprenoid quinones while maintaining lateral mobility. A pigment-dependent bias of the fluorescence results could be excluded. The examined pigments were characterized by an absorption spectrum with a single maximum between 450 and 460 nm. This resulted in approximately equal absorption intensities at the two wavelengths for determining Laurdan-GP values. The possible result bias by selective absorption of polarized light was compensated by determination of the G-factor for each temperature value and approach.

Moreover, we could demonstrate that carotenoid enrichment in the cells increased resistance against freeze-thaw stress and therefore had a protective effect on the cell at low temperatures. We found an only marginal log reduction in viable cell counts for cultures grown at 10 °C with elevated carotenoid concentrations. In contrast, cultures of both strains grown at 30 °C showed clearly higher log reduction of viable cell counts after up to three freeze-thaw cycles, resulting in a >99% cell loss for strain J70 and >90% for strain DSM 20266^T^ (Fig. 4). To confirm the impact of carotenoid content on membrane fluidity regulation and cold survivability, we suppressed carotenoid synthesis in *S. xylosus* J70 at low temperatures. In a first approach, we inhibited carotenoid synthesis by diphenylamine (DPA) supplementation according to Hammond & White^46^. We successfully produced non-pigmented cells at a DPA concentration of 74 µM. However, this approach turned out to be inappropriate because DPA accumulates in the cell membrane and substantially altered membrane properties. An alternative approach was to suppress carotenoid synthesis by NaCl supplementation as described for *Arthrobacter agilis* by Fong *et al*.^27^. Supplementation with 5% NaCl resulted in unpigmented cells at the point of cell harvest. Carotenoid concentration was below the detection limit for these cells. For strain DSM 20266^T^, complete inhibition could only be observed above 10% NaCl. However, this concentration had a clearly reducing effect on growth rates, so we focused on the supplementation of strain J70 with 5% NaCl. This strain showed no decrease of grow rates with 5% NaCl but complete loss of pigmentation. The addition of NaCl had no significant effect on the fatty acid profile, which can be derived from the WAMT values (Table 1). The probe-dependent measurement data showed that membrane fluidity as well as membrane order of the unpigmented cultures grown at 10 °C were almost identical to the cultures grown at 30 °C (Fig. 3). Cultures grown at 30 °C with and without NaCl-supplementation showed no impact of the salt stress on membrane parameters. These results confirmed that the observed effects on membrane properties were actually caused by the carotenoid content of the cells. The same was true for the increased resistance to freeze-thaw stress, which could be attributed to the carotenoid content of the cells. The NaCl-supplemented cultures of strain J70 grown at 10 °C showed almost identical log reduction as the cultures grown at 30 °C. Consequently, the improved cold survival seems to be derived from the carotenoids and not from general cold adaptation during growth at low temperatures, in this case.

To our knowledge, this work demonstrates for the first time the modification of membrane fluidity and membrane order in living cells by increasing the carotenoid content of the cell. This improves membrane fluidity at low temperatures, and is in accord to previous findings in artificial liposomes. In addition, the cell viability is improved under low temperature conditions. The increase of the transcriptional level of genes involved in carotenoid biosynthesis identified this mechanism as integral part of the controlled and regulated adaptive response to low ambient temperature.

## Materials and Methods

### Strains, culture media and cultivation

*Staphylococcus xylosus* strain J70 was isolated from raw milk taken from a refrigerated milk tank in the year 2011. Strain J70 was not pigmented at 30 °C growth temperature, but a distinct yellow pigmentation became visible at 10 °C growth temperature. Fatty acid analysis and 16 S rRNA gene sequencing was used to identify the species. Type strain *S. xylosus* DSM 20266 ^T^ was included as a reference strain in this study.

*S. xylosus* strains were grown aerobically (agitated at 120 rpm) in CASO-Bouillon (Merck 105459), composed of peptone from casein (17.0 g/l), peptone from soymeal (3.0 g/l), D(+)glucose (2.5 g/l), sodium chloride (5.0 g/l; NaCl), di-potassium hydrogen phosphate (2.5 g/l). Carotenoid synthesis was inhibited by additional NaCl supplementation, as previously described by Fong *et al*.^27^. In this study CASO-Bouillon was supplemented with 50 g/l NaCl resulting in a total sodium chloride concentration of 5.5%. Cultures were prepared in triplicates, inoculated with 1% (v/v) overnight culture and incubated at 30 °C or 10 °C in the dark until late exponential phase (A_625_ = 0.8–1). Cell densities were measured at 625 nm with a UV-Vis spectrophotometer (Genesys 10uv Scanning; Thermo Fisher Scientific, Waltham, USA). Cultures were harvested by centrifugation at the corresponding growth temperatures, 12,860 × *g* for 10 min and washed twice with pre-tempered 137 mM phosphate buffered saline (PBS), pH 7.4. Afterwards cells were used for fatty acid analysis, menaquinone analysis, carotenoid analysis and membrane fluidity measurements. Cell colonies were cultivated on CASO-Agar (Merck 105458) at 30 °C.

### Temperature stress tests

A temperature stress test was performed by subjecting each strain to three freeze-thaw cycles. Cells grown at 30 °C or 10 °C were aliquotted into 3 × 2 ml cell suspensions for each strain and were frozen at −20 °C. After 24, 48, and 72 h cells were thawed for 10 minutes at room temperature and the viable cell count was determined. For this purpose, a decadal dilution series (1 in 9 ml) was prepared in sterile Ringer’s solution and plated onto TSA plates. After one day incubation at 30 °C, the colonies were counted for the corresponding dilution steps and the weighted mean of cell counts were provided in cfu/ml. The remaining sample volume was re-frozen for subsequent freeze-thaw cycles.

### Fatty acid analysis

Fatty acids were extracted by methanolysis as described by Sasser^47^. Approximately 50–100 mg cell material per sample was used for fatty acid analysis. Cells were resuspended in 1.0 ml 15% NaOH in methanol/bidest. water 1:1 (v/v) and saponified for 30 min at 100 °C. Fatty acids were methylated with 2.0 ml 6.00 N HCl/Methanol, 13:11 (v/v) for 10 min at 80 °C, and rapidly cooled in an ice bath. Fatty acid methyl esters were than extracted with 1.25 ml hexane/methyl-tert-butyl-ether (MTBE), 1:1 (v/v) for 10 min in an overhead shaker. Phases were separated by centrifugation for 5 min at 1160 × *g* and the lower phase was discarded. Subsequently, a base wash of the upper phase was performed with 3.0 ml of 1.2% NaOH in bidest. water. Fatty acid methyl ester identification was performed with a GC-MS using a gas chromatograph (model 7890 A; Agilent Technologies Germany GmbH, Waldbronn, Germany) equipped with a 5% phenyl methyl silicone capillary column and a coupled mass spectrometer (model 5975 C; Agilent Technologies Germany GmbH) as previously described by Lipski and Altendorf^48^. ChemStation software (Agilent Technologies Germany GmbH) was used to analyse GC-MS data and fatty acids were identified by their retention time and mass spectra.

The magnitude of fatty acid adaptation to low temperature was deduced from weighted average melting temperature (WAMT; Eq. 1;^22^). Considering the individual melting temperatures of each fatty acid this parameter allows us to integrate the quantative changes of all membrane fatty acids. The WAMT value does not represent the actual melting temperature of the cytoplasmic membrane, which also depends on the total polar lipid structure. Melting temperatures for all fatty acids are taken from Knothe and Dunn^49^.

1 $$\begin{array}{ccc}{\rm{W}}{\rm{A}}{\rm{M}}{\rm{T}} & = & {\rm{P}}{\rm{e}}{\rm{r}}{\rm{c}}{\rm{e}}{\rm{n}}{\rm{t}}{\rm{a}}{\rm{g}}{\rm{e}}({{\rm{F}}{\rm{A}}}_{1})\times {{\rm{T}}}_{{\rm{m}}}({{\rm{F}}{\rm{A}}}_{1})+{\rm{P}}{\rm{e}}{\rm{r}}{\rm{c}}{\rm{e}}{\rm{n}}{\rm{t}}{\rm{a}}{\rm{g}}{\rm{e}}({{\rm{F}}{\rm{A}}}_{2})\times {{\rm{T}}}_{{\rm{m}}}({{\rm{F}}{\rm{A}}}_{2})+\ldots \\  &  & +{\rm{P}}{\rm{e}}{\rm{r}}{\rm{c}}{\rm{e}}{\rm{n}}{\rm{t}}{\rm{a}}{\rm{g}}{\rm{e}}({{\rm{F}}{\rm{A}}}_{{\rm{n}}})\times {{\rm{T}}}_{{\rm{m}}}({{\rm{F}}{\rm{A}}}_{{\rm{n}}})\end{array}$$

FA_1_ to FA_n_ are all fatty acids present in the fatty acid profile, percentage(FA_1_) is the percentage of fatty acid no. 1 and T_m_(FA_1_) is the melting temperature of this fatty acid. The difference in WAMT at both growth temperatures (ΔWAMT) indicates the extent of cold adaptation by means of fatty acid shift.

### Isoprenoid quinone analysis

Wet cells were used for a chloroform extraction of isoprenoid quinones as already described by Seel *et al*.^22^. Pellets between 20 and 100 mg cell wet weight were suspended in 3 ml 48 mM K_2_HPO_4_ (pH 7.4) buffer using 50 ml hydrolysis tubes. Nine ml methanol and 5 ml chloroform containing an internal vitamin K_1_ (Sigma-Aldrich 95271) standard were added to the suspended cells and shaken overhead for 30 min. For phase separation 5 ml bidest. water and 5 ml chloroform were added and centrifuged at 1160 × *g* for 5 min. Chloroform extraction was repeated twice, organic phases were pooled and evaporated to dryness using a rotary vacuum evaporator (100 mbar, 38 °C; IKA Werke GmbH & Co. KG, Staufen, Germany). Residues were dissolved in 5 ml hexane. Crude extracts were purified by using solid phase extraction columns^50^. Quinone extracts were analysed on an Agilent 1260 HPLC series consisting of a Quat Pump, auto sampler, thermo-controlled column compartment, and a diode array detector. Compounds were separated isothermally at 30 °C on a reversed-phase column (ODS Hypersile RP18, Thermo Fisher, USA). Mobile phase consisted of a methanol/isopropyl ether mixture (9:2 v/v) and the flow rate was set to 1 ml/min. Isoprenoid quinones were detected at 270 nm and 275 nm and were identified by their absorption spectrum and retention time. The identification was based on known quinone patterns of different type strains. The concentration was calculated using an internal standard of 6.55 nmol vitamin K_1_^22^.

### Carotenoid analysis

Carotenoid extraction was performed according to Kaiser *et al*.^51^. This method includes by a combination of different enzymatic and mechanical cell disruption processes, which ensure a mild as well as complete extraction of carotenoids. A maximum of 35 mg of the lyophilized cells were used to achieve complete extraction from the dry cell mass and were transferred to 2 ml reaction vessels. For enzymatic cell lysis, cells were resuspended in 1.2 ml ice-cold PBS (137 mM NaCl, 12 mM H_2_HPO_4_, 2.7 mM KCl, pH 7.4) and vortexed after addition of 200 µl lipase PBS-solution (2 U) and 200 µl lysostaphin (2,7 kU) for 10 s. The samples were incubated in the dark in a shaking incubator at 37 °C, 250 rpm for 2 hours. Subsequently, a combined mechanical cell disruption consisting of freeze-thaw cycles and an ultrasound bath treatment were performed. Samples were frozen three times at −80 °C (5 to 10 min), then thawed in a water bath (Julabo MP-BRÜ/PU, Seelbach, Germany) at 30 °C and then treated for 3 min in a cooled ultrasonic bath (Sonorex, BANDELIN electronic, Berlin, Germany). Analysis was performed by HPLC-DAD-atmospheric pressure chemical ionization mass spectrometry as previously described by Etzbach *et al*.^52^. Samples were dissolved in 1 ml methanol/MTBE mixture (1:1 (v/v), stabilized with 0.1% butylhydroxytoluene). The autosampler was tempered to 15 °C and the column oven to 25 °C. A combination of a precolumn Accucore™ C30 (10 × 2.1 mm, 2.6 µm; Thermo Fisher Scientific) and the column Accucore™ C30 (150 × 2.1 mm, 2.6 µm; Thermo Fisher Scientific) was used. As an external standard, β-carotene (Extrasynthese, Lyon, France), with a purity > 99%, was used for quantitative and qualitative analysis. The injection volume was 5 µl. Elution was performed with a gradient of methanol, water and MTBE and a flow rate of 0.4 ml/min^52^. The mass spectrometric determination was performed in a range from *m/z* 100 to 1200. The software used was Xcalibur 2.2 SP1.48 from Thermo Fisher Scientific (Schwerte, Germany). The set parameters of the mass spectrometer were described in detail previously^52^.

### Membrane fluidity analyses by general polarization and anisotropy

Whole cells were stained with two different fluorescent probes in order to determine membrane order and membrane fluidity properties^22,45^. Based on the polar relaxation of the Laurdan-molecule, primarily the degree of order of the adjacent lipids is measured by Laurdan-GP measurement. High Laurdan-GP values correspond to a high membrane order and low GP values to a low membrane order. TMA-DPH anisotropy is particularly suitable for measuring membrane fluidity. In this case the direct mobility of the probe and the adjacent lipids is measured. Steady-state fluorescence was measured in a LS 55 spectrofluorometer combined with a Peltier element PTP-1 (PerkinElmer LAS GmbH, Rodgau, Germany) for sample temperature regulation. Sample preparation was done as described by Seel *et al*.^22^. Cells of the late-exponential growth phase were washed and suspended in 48 mM K_2_HPO_4_ (pH 7.4) buffer and diluted to an optical density of 0.2 at 625 nm. Laurdan stock solution was prepared in ethanol at 2 mM and stored in the dark at 4 °C. Staining was performed at a concentration of 20 µM for 30 min at 30 °C in the dark. Calibration curves of the lipid to dye ratio were established between 1 and 60 µM in order to ensure stable GP-values. Stable GP-values between 5 and 40 µM were present for the cell density used. TMA-DPH stock solution was prepared in DMSO with a concentration of 400 µM. Cells were stained with 0.5 µM TMA-DPH for 30 min at 30 °C in the dark. According to Laurdan, calibration curves for TMA-DPH in the range of 0.1 to 10 µM were determined, with stable anisotropy values throughout. For every temperature step cells were equilibrated for 2 min.

In the present study, incorporation time of the TMA-DPH probe was increased to 30 min to improve probe integration into bacterial membranes. Two ml sample volume in 3.5 ml quartz cuvettes (Hellma GmbH, Müllheim, Germany) were used for measurements. For Laurdan GP analyses samples were excitated at 360 nm (slit 10.0 nm) at each temperature step and the emission spectra (slit 3.0 nm) were recorded from 380 nm to 600 nm. GP values were calculated using the emission intensity values at 435 nm and 500 nm (^45^; Eq. 2):

2 $${\rm{GP}}=\frac{{I}_{435}-{I}_{500}}{{I}_{435}+{I}_{500}}$$

For TMA-DPH anisotropy measurements samples were excitated at 355 nm and emission intensities recorded at 425 nm. Anisotropy values were calculated from polarized intensities using the following equation:

3 $$r=\frac{{I}_{{\rm{VV}}}-G{I}_{{\rm{VH}}}}{{I}_{{\rm{VV}}}+2G{I}_{{\rm{VH}}}}$$

where *I* is the fluorescence intensity from which blank values from non-labeled cells were subtracted. *G* stands for *G*-factor, calculated by the ratio of *I*_HV_/*I*_HH_. The subscripts H (horizontal) und V (vertical) indicate the polarizer positions for the excited and the emitted light. Each data point was calculated from 10–20 single measurements. Data are shown as means with standard deviations from independent biological triplicates.

### Differential transcriptome analyses

For mapping of transcribed genes a draft genome sequence was generated for strain *S. xylosus* J70. Total DNA was isolated using a Qiagen DNeasy Blood and Tissue Kit with subsequent RNase treatment according to the manufacturer’s instructions. Genome sequencing was performed using an Illumina HiSeq. 3000 platform (Illumina, San Diego, USA) sequencer. For library preparation NEB Next Ultra II DNA Library Kit for Illumina (New England Biolabs) was used. Fragments with a read length of 150 bp paired ends were sequenced resulting in a minimum of 3,333,333 reads (equivalent to 1.00 Gigabases). Reads were checked for sufficient quality and adapters were trimmed with FLEXBAR^53^. Genomic assembly was done with SPAdes-3.6.2^54^ and assessed with Quast^55^. Coverage of the assembled contigs was calculated with the software bbmap as part of the BBTools suite (BBMap – Bushnell B. – sourceforge.net/projects/bbmap/). Overlaps and similarities of the contigs with a size >500 bp were checked with Gepard^56^. For gene prediction and annotation the RAST-server (rapid annotation using subsequent technology) was used^57^.

For gene expression analyses cells were cultivated at 10 °C and 30 °C, respectively. Cells were harvested at the late exponential stage and mixed with RNAprotect-Bacteria (Qiagen, Hilden, Germany) to stabilize RNA. Total RNA was isolated using a Qiagen RNeasy Protect Bacteria Mini Kit with subsequent DNase treatment and eluted in RNase-free water according to the manufacturer’s instructions. RNA depletion was performed to remove the high amounts of rRNA and maximize encoding RNA. Fragments with a read length of 150 bp paired ends were sequenced with Illumina HiSeq. 3000 resulting in a minimum of 5,000,000 reads (equivalent to 1.50 Gigabases). Alignment of the mRNA reads was done with Bowtie^58^ and TopHat2^59^. The read counts were calculated with HTSeq-count^60^. Subsequently, the mRNA reads were mapped on the previously created genome assembly using EdgeR software^61,62^. Differential gene expressions were calculated with EdgeR using default settings of the program. The calculations were based on the gene coverage, statistical parameter p-value, false discovery rate (FDR), and Bonferroni-value. For an EdgeR analysis, the data structure is analysed based on a negative binomial model including normalization factors and dispersion values to ensure comparability. The differently expressed genes were selected according to their significance in Chi-square tests and at least 2-fold differences.

### Statistical evaluation

Mean values of triple biological replicates were calculated for all experiments; error bars represent the corresponding standard deviation.

### Significance statement

Carotenoids are associated with several important biological functions as antenna pigments in photosynthesis or protectives against oxidative stress. Here we could demonstrate that carotenoids are also involved in the adaptive response of the bacterial membrane to low temperature and the regulation of membrane fluidity. Moreover, carotenoids caused an increased resistance against freeze-thaw stress. Carotenoid synthesis is transcriptionally upregulated under low temperature conditions. Therefore, carotenoid synthesis can be considered as integral part of the controlled and regulated adaptive response to low temperature conditions.
